# Author Correction: Shear band-driven precipitate dispersion for ultrastrong ductile medium-entropy alloys

**DOI:** 10.1038/s41467-021-26008-1

**Published:** 2021-09-23

**Authors:** Tae Jin Jang, Won Seok Choi, Dae Woong Kim, Gwanghyo Choi, Hosun Jun, Alberto Ferrari, Fritz Körmann, Pyuck-Pa Choi, Seok Su Sohn

**Affiliations:** 1 Department of Materials Science and Engineering Korea University, Seoul, South Korea; 2 Department of Materials Science and Engineering Korea Advanced Institute of Science and Technology, Daejeon, South Korea; 3 Center for High Entropy Alloys Pohang University of Science and Technology, Pohang, South Korea; 4 Department of Materials Science and Engineering Delft University of Technology, Mekelweg 2, Delft, The Netherlands; 5 Max-Planck-Institut für Eisenforschung Max-Planck-Straße 1, Düsseldorf, Germany

**Keywords:** Mechanical properties, Metals and alloys

Correction to: *Nature Communications* 10.1038/s41467-021-25031-6, published online 4 August 2021.

The original version of this Article omitted an equal contribution statement for Tae Jin Jang and Won Seok Choi. This has now been corrected in both the PDF and HTML versions of the Article.

