# Supplementary material for: Shear band-driven precipitate dispersion for ultrastrong ductile medium-entropy alloys
Source: Nat Commun. 2021 Aug 4;12:4703. doi: 10.1038/s41467-021-25031-6 (PMC8338972; doi:10.1038/s41467-021-25031-6)
Supplement: Supplementary file 1 — Supplementary Information [file 41467_2021_25031_MOESM1_ESM.pdf]

Supplementary information for

Shear band-driven precipitate dispersion for ultrastrong ductile medium-entropy alloys

Tae Jin Jang [1], Won Seok Choi [2], Dae Woong Kim [3], Gwanghyo Choi [2], Hosun Jun [2], Alberto Ferrari [4], Fritz Körmann [4,5], Pyuck-Pa Choi [2, *], and Seok Su Sohn [1, **]

[1] Department of Materials Science and Engineering
Korea University, 02841 Seoul, South Korea

[2] Department of Materials Science and Engineering
Korea Advanced Institute of Science and Technology, 34141 Daejeon, South Korea

[3] Center for High Entropy Alloys
Pohang University of Science and Technology, 37673 Pohang, South Korea

[4] Department of Materials Science and Engineering
Delft University of Technology, Mekelweg 2, 2628 CD Delft, The Netherlands

[5] Max-Planck-Institut für Eisenforschung
Max-Planck-Straße 1, 40237 Düsseldorf, Germany

* Corresponding authors: p.choi@kaist.ac.kr; sssohn@korea.ac.kr

Supplementary Note. The details of phase stability calculations. To investigate the precipitation reactions in $Al_{0.2}CoNiV$, DFT calculations were conducted for the relative phase stability of the ordered phases $L1_2$-$(Co,Ni)_3Al$, B2-(Co,Ni)Al, and $L2_1$-$(Co,Ni)_2VAl$. The phase stability is determined by the free energy of both the precipitate and the matrix, whose composition balances that of the precipitate. The construction of a multi-dimensional convex hull for the free energy of $Al_{0.2}CoNiV$ with DFT is impractical in the multicomponent phase space and thus beyond the scope of the present work. To provide an appropriate comparison of the candidate precipitates at reasonable computational costs, two approximations were made: (1) to fix the compositions and phase fractions of the precipitate and matrix, all available Al (6.25 at%) was assumed to be sequestered by the precipitates, i.e., the matrix was completely depleted of Al; and (2) the lattice parameters were determined such that a Wigner–Seitz radius of 1.411 Å was obtained for every structure, corresponding to the computed 0 K equilibrium volume of the initial FCC $Al_{0.2}CoNiV$ solid solution.

For the $L2_1$ phase, different populations of the (Co,Ni) lattice were considered, with a Co concentration in the sublattice ranging from 0 to 100%. For each configuration, the first bar corresponds to the energy difference at 0 K in the ferromagnetic state, the second in the paramagnetic DLM state (+magn), while the third adds the electronic and configurational contributions (+el, +conf) at 1150 K (within the annealing temperature window). In agreement with the experiments, the most stable precipitate among the candidates was determined to be the Co-rich $L2_1$ phase.

From an electronic-structure viewpoint, the stabilization of this Co-rich phase originates from the opening of a pseudogap in the electronic density of states of the $L2_1$ phase in proximity to the Fermi level (E_F). The larger the Co concentration, the closer the pseudogap to E_F; hence, the more stable the electronic configuration at 0 K. The ferromagnetic simulations at 0 K predicted that, for the most stable configuration, Ni was completely absent, whereas the APT analysis and experimental lattice parameter rather suggest a Co and Ni ratio of approximately 2:1. Notably, however, if finite-temperature contributions are included (magnetic disorder and configurational and electronic free energy at 1150 K), the $L2_1$ phases with 67%, 75%, and 100% Co become almost degenerate (free energy difference less than 5 meV/at.), and with a further

increase in the temperature, a progressively larger Ni content becomes energetically favorable in the $L2_1$ phase, in good qualitative agreement with the experiments.

The different free energy contributions can be understood as follows: (1) the pseudogap being closest to E_F for the $L2_1$-Co phase also causes a smaller electronic free energy contribution due to the less available electronic states near E_F with respect to the $L2_1$-(Co,Ni) phase; (2) paramagnetism shifts the Fermi energy of the $L2_1$-Co phase away from the pseudogap; and (3) configurational entropy in the ideal mixing favors a random (Co,Ni) sublattice in the $L2_1$ phase, thereby further stabilizing a partial Ni segregation in the $L2_1$ phase. Given the delicate balance of the individual contributions and the overall small energy differences, the inclusion of other contributions (e.g., lattice vibrations, short-range order, elastic contributions, and relaxation effects) may provide a more quantitative agreement with the experimentally observed composition. It is also confirmed that the results remain qualitatively unaltered when V partially occupies the Al sublattice in the $L2_1$ phase (not shown here).

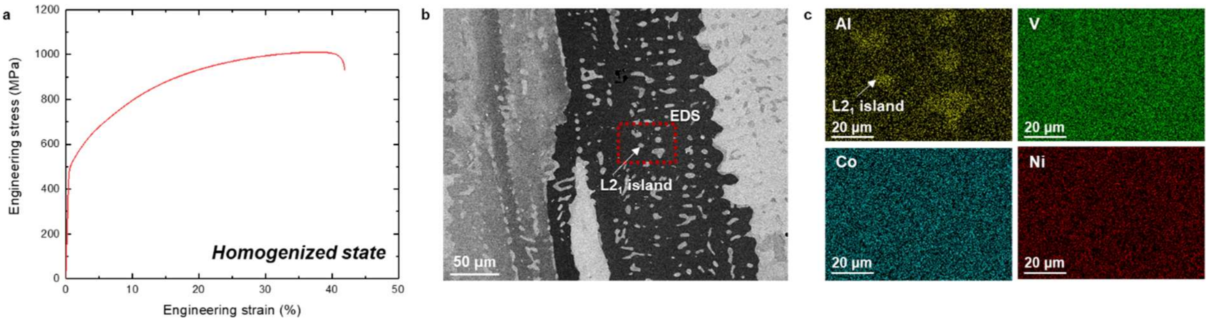

Supplementary Fig. 1 a Tensile stress-strain curve of the homogenized state, **b, c** SEM image and corresponding EDS elemental maps exhibiting $L2_1$ islands.

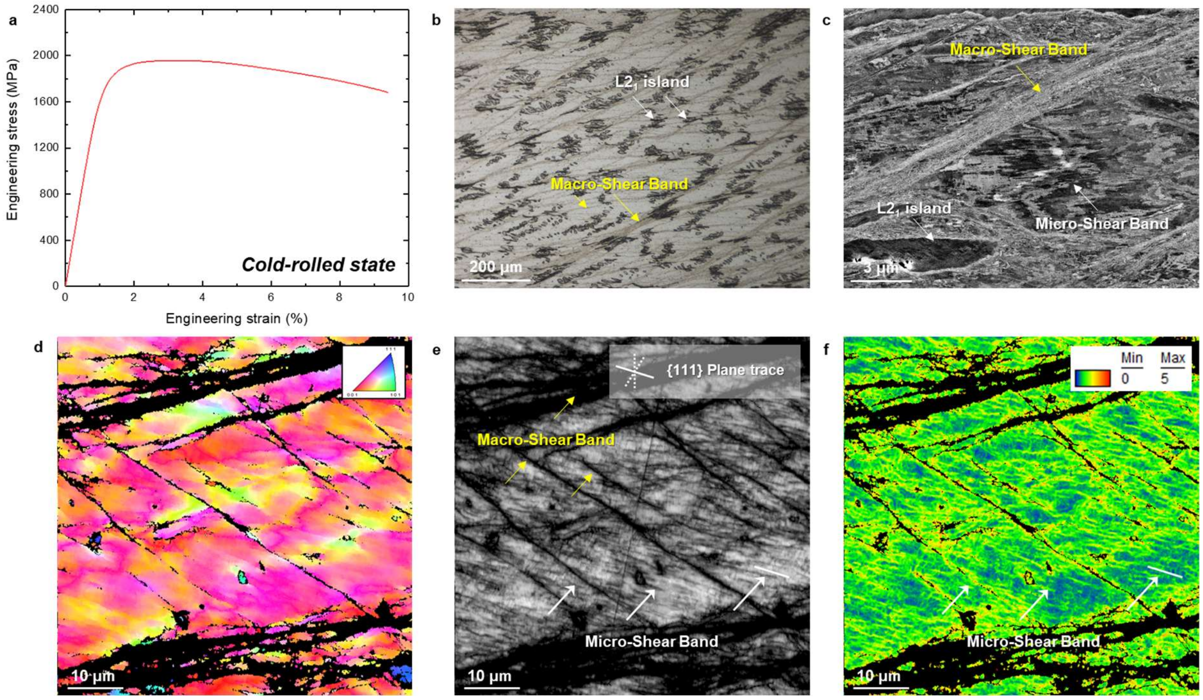

Supplementary Fig. 2 a Tensile stress-strain curve of the cold-rolled state, **b, c** optical and SEM images showing macro-shear bands along ~10–45 degrees to the rolling direction, **d-f** EBSD IPF, IQ, and KAM maps showing macro-shear bands and micro-shear bands indicated by yellow and white arrows, respectively.

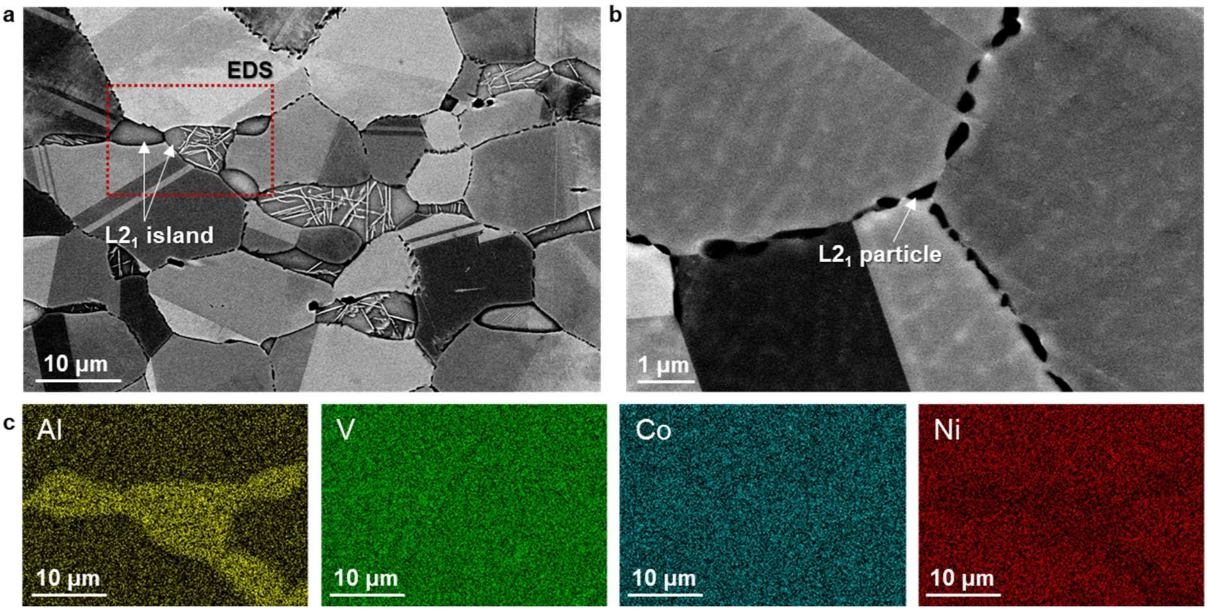

Supplementary Fig. 3 a, b Low- and high- magnification SEM images, and **c** EDS elemental maps exhibiting L2$_1$ island of the RA sample.

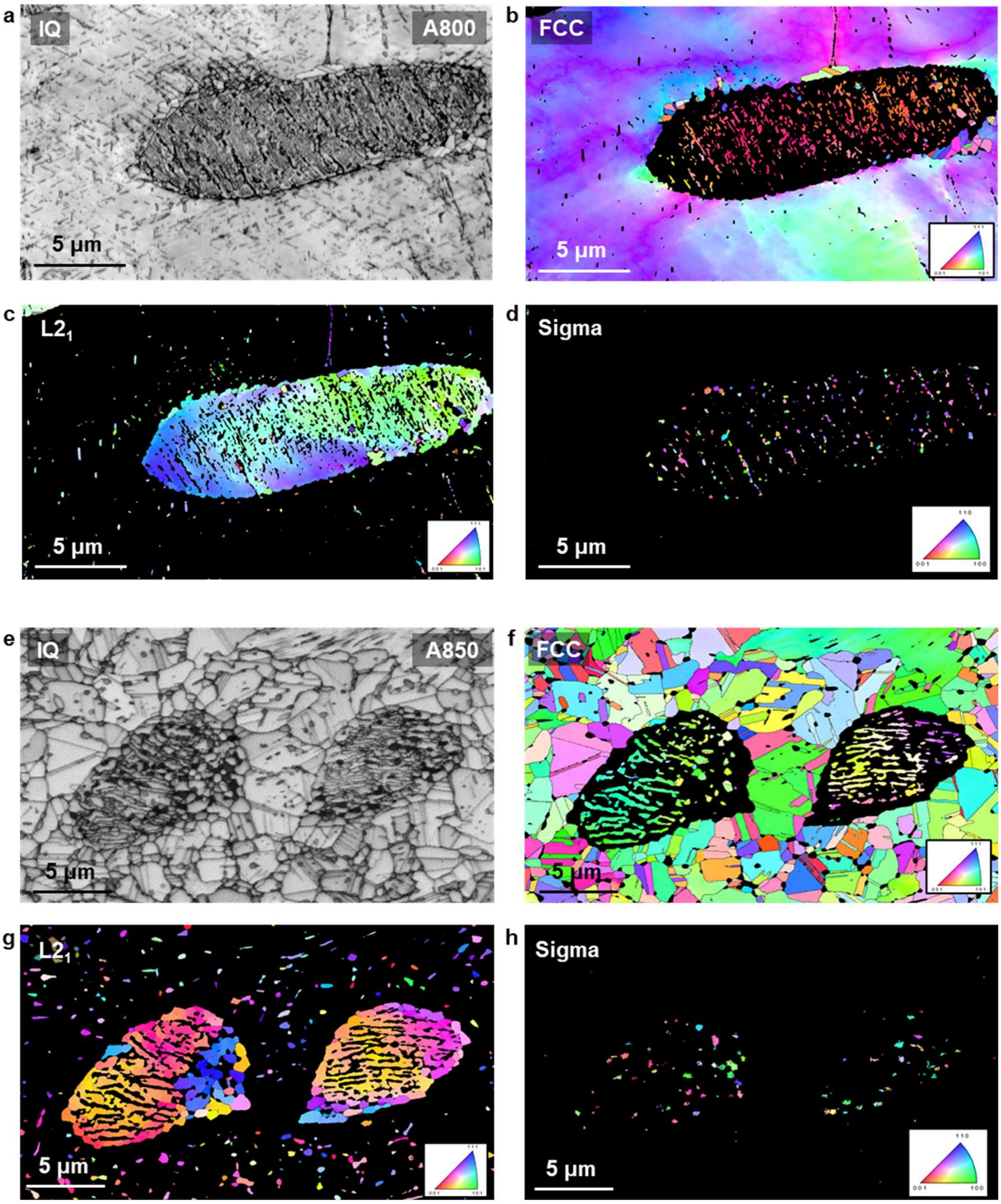

Supplementary Fig. 4 Microstructures revealed by EBSD analysis for the annealed $Al_{0.2}$CoNiV alloy. a EBSD IQ map, **b** IPF map of FCC, **c** IPF map of L21, and **d** IPF map of sigma phase for the alloy annealed at 800 °C for 1 h. **e** EBSD IQ map, **f** IPF map of FCC, **g** IPF map of L21, and **h** IPF map of sigma phase for the alloy annealed at 850 °C for 1 h.

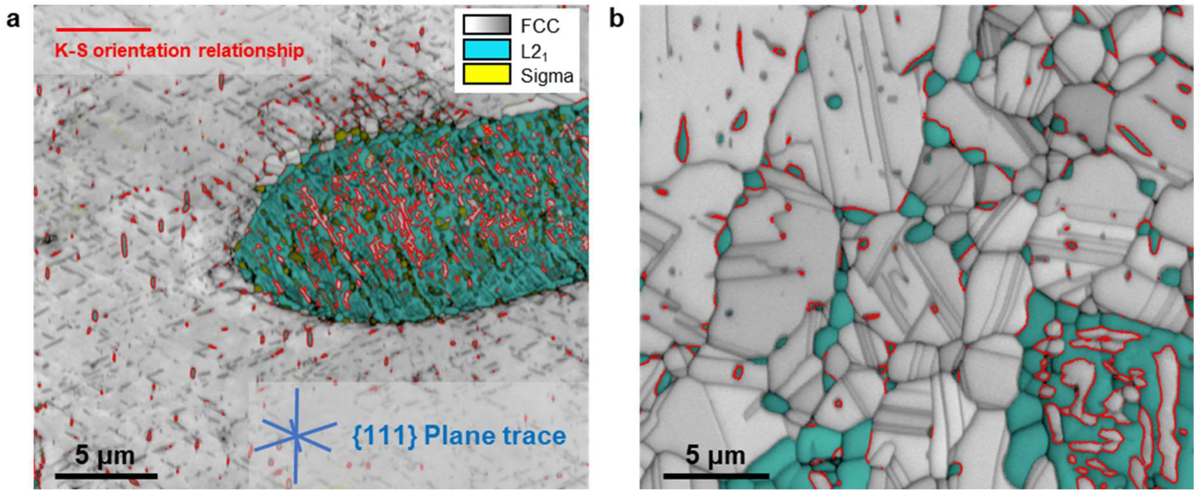

Supplementary Fig. 5 EBSD IQ maps of annealed $Al_{0.2}CoNiV$ alloys superimposed by phase color. White-gray, turquoise, and yellow colors represent FCC, $L2_1$, and σ phases, respectively. **a** non-recrystallized region of the alloy annealed at 800 °C and **b** recrystallized region of the alloy annealed at 900 °C. Red line indicates FCC and $L2_1$ phases having a K-S orientation relationship.

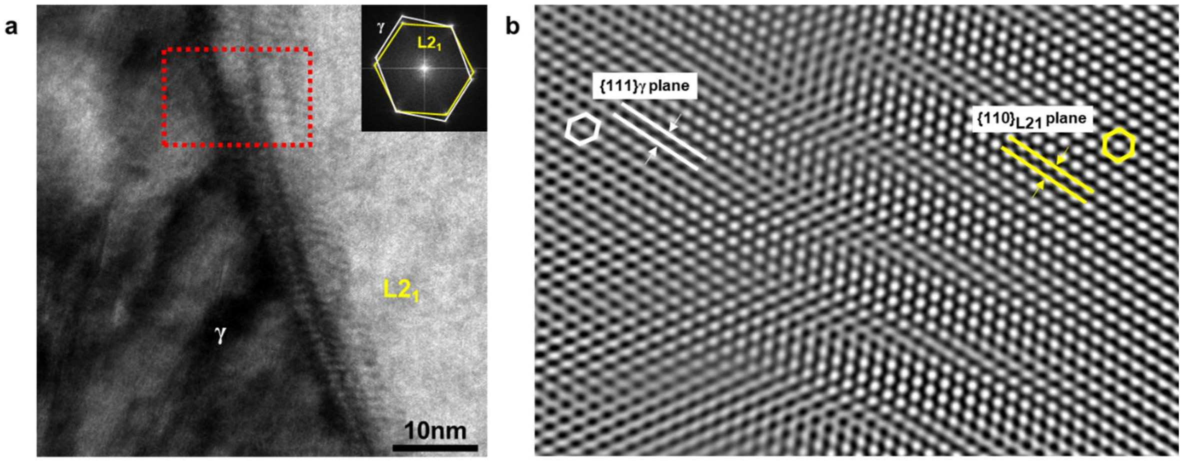

Supplementary Fig. 6 a High-resolution TEM and corresponding FFT images, **b** inverse FFT image showing semi-coherent interface between γ and $L2_1$.

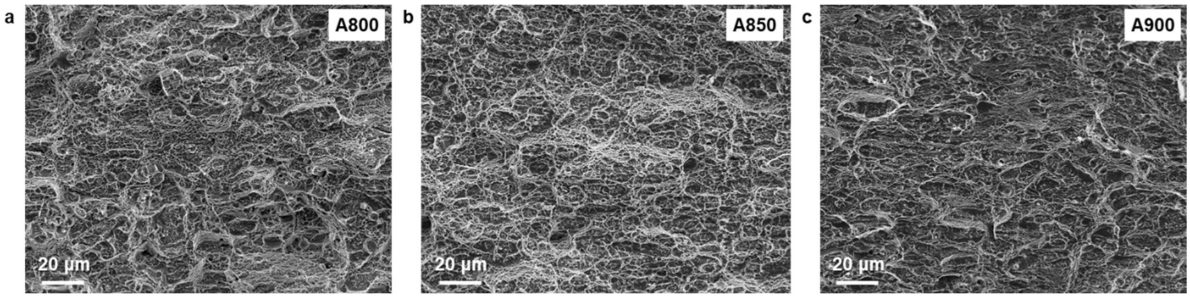

Supplementary Fig. 7 Tensile fractographies of the **a** A800, **b** A850, and **c** A900 alloys. Only ductile-dimple fracture was observed.

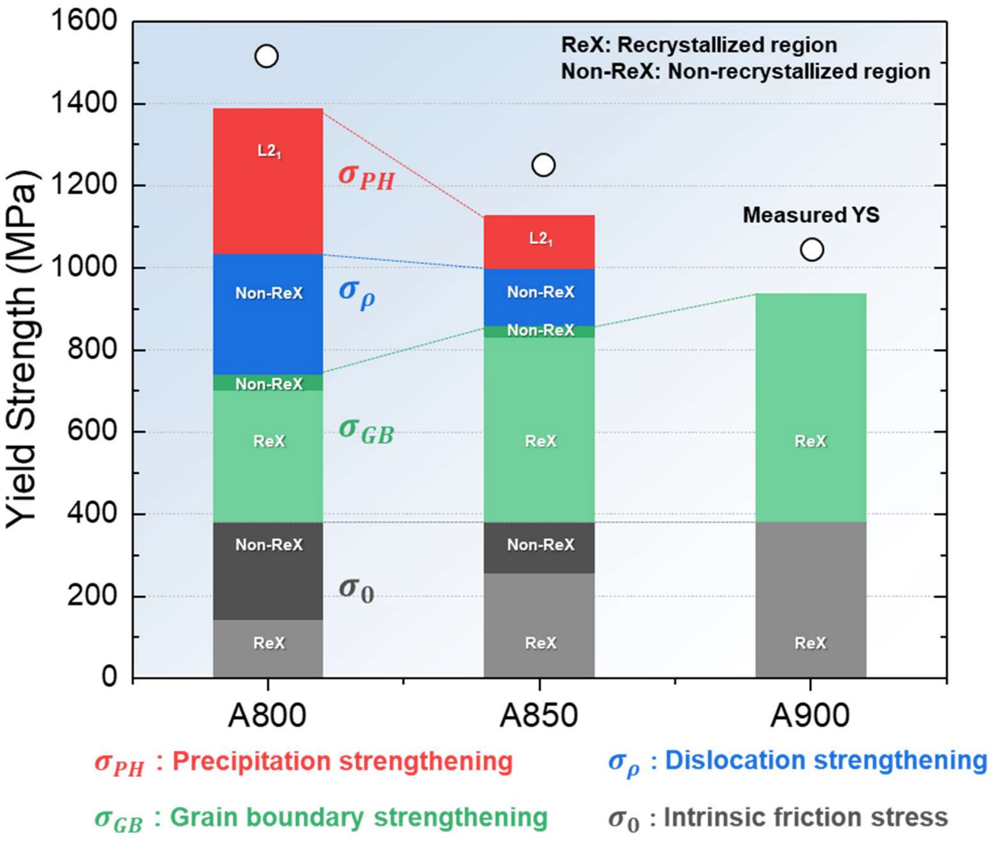

Supplementary Fig. 8 Summarized chart showing calculated strengthening contributions from each mechanism for alloys annealed at 800, 850, and 900 °C for 1 h.

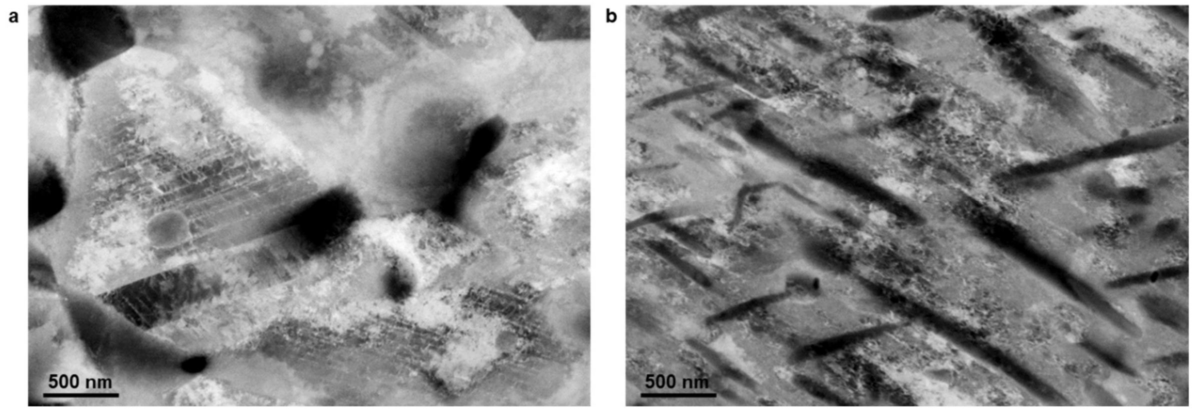

Supplementary Fig. 9 ECCI micrographs of the $Al_{0.2}$CoNiV alloy annealed at 850 °C after tensile test at room temperature. **a** recrystallized region, **b** non-recrystallized region.

Supplementary Table 1. Fractions of constituent phases for the $Al_{0.2}CoNiV$ alloy annealed under three conditions (%).

Region		A800	A850	A900
$L2_1$ island	Total	11.3 ± 2.6	9.6 ± 3.7	9.6 ± 3.0
	FCC	5.5 ± 0.5	4.0 ± 0.2	2.8 ± 0.3
	$L2_1$	4.8 ± 0.4	5.2 ± 0.2	6.8 ± 0.3
	σ	1.0 ± 0.1	0.4 ± 0.1	0
Recrystallized	Total	33.9 ± 2.7	60.7 ± 3.5	90.4 ±3.0
	FCC	27.8 ± 0.4	53.4 ± 0.9	83.9 ± 0.2
	$L2_1$ at GB*	5.6 ± 0.4	6.3 ± 0.5	5.4 ± 0.1
	$L2_1$ at IG**	0.5 ± 0.1	1.1 ± 0.3	1.1 ± 0.3
Non-Recrystallized	Total	54.8 ± 5.0	29.7 ± 4.0	0
	FCC	46.0 ± 1.3	25.3 ± 0.2	0
	$L2_1$ at SB***	8.8 ± 1.3	4.40 ± 0.2	0

*GB: Grain boundary, **IG: Inside grain, ***SB: Shear band

Supplementary Table 2. Sizes of FCC, $L2_1$, and σ phases for the $Al_{0.2}CoNiV$ alloy annealed under three conditions.

Region	Phase	A800	A850	A900
$L2_1$ island	$L2_1$ (μm)	7 ± 4	10 ± 8	8 ± 6
	FCC (nm)	40 ± 20	200 ± 90	300 ± 100
	σ (nm)	120 ± 80	110 ± 80	
Recrystallized FCC	FCC (μm)	1.1 ± 0.7	1.7 ± 0.9	2 ± 2
	$L2_1$ at GB* (nm)	210 ± 10	200 ± 200	300 ± 200
	L21 at IG** (nm)	90 ± 3	143 ± 6	146 ± 6
Non-Recrystallized	FCC (μm)	210 ± 70	90 ± 20	
	$L2_1$ at SB***(nm)	57 ± 7	90 ± 30	

*GB: Grain boundary, **IG: Inside grain, ***SB: Shear band

Supplementary Table 3. Room-temperature tensile properties of the $Al_{0.2}CoNiV$ alloys.

Specimen	Yield Strength (MPa)	Tensile Strength (MPa)	Elongation (%)
A800	1500 ± 10	1730 ± 20	8 ± 1
A850	1260 ± 10	1590 ± 10	27 ± 3
A900	1050 ± 20	1480 ± 10	32 ± 4
RA	570 ± 8	1120 ± 10	49 ± 2

Supplementary Table 4. Compositions, processing, constituent phases, grain size and tensile properties of the MEAs and HEAS reported previously.

Alloys	Processing	Phases	GS (μm)	Strain rate (s^{-1})	YS (MPa)	UTS (MPa)	U. EL (%)	T. EL (%)	Ref.
$Al_{0.1}CoCrFeNi$	As-received	FCC	few mm	1×10^{-3}	160	389	44.0	46.2	1
	FSP	FCC	0.35-13.5	1×10^{-3}	544	730	27.5	56.0	1
$Al_{0.25}CoCrFeNi$	AC	FCC		5×10^{-4}	118	807	55.2	55.2	2
$Al_{0.3}CoCrFeNi$	AC + CR (50%) + 1000°C/10h/SC	FCC		5×10^{-4}	150	758	51.1	51.1	2
	AC + 700°C/72h/WQ	FCC+$L1_2$		4×10^{-4}	310	525	43.0	44.0	3
	AC + 900°C/72h/WQ	FCC+B2		4×10^{-4}	240	570	42.0	45.0	3
	AC	FCC		4×10^{-4}	275	528	31.5	37.0	4
	AC + 1250°C/50h/SC + 1250°C UF (50%)	FCC		2×10^{-4}	210	500	88.0	97.0	5
	AC + 1150°C/1h/WQ + CR (50%) + 800°C/50h	FCC+B2		1×10^{-3}	702	1002	28.0	33.0	6
	AC + hot forging (1050°C) +hor-drawing (900°C)	FCC+B2		1×10^{-3}	1147	1207	12.0	12.0	7
$Al_{0.5}CoCrFeNi$	AC	FCC+BCC		1×10^{-3}	355	714	41.0	41.6	8
	AC + 650°C/8 h/WQ	FCC+BCC+B2		1×10^{-3}	834	1220	25.0	26.0	8
$Al_{0.7}CoCrFeNi$	AC + 1250°C/50h/SC + 1250°C UF (50%)	FCC+BCC/B2		2×10^{-4}	600	740	8.0	8.0	5
$AlCoCrFeNi_{2.1}$	AC	FCC+$L1_2$+B2		8.3×10^{-4}	620	1050	16.0	17.0	9
	AC + CR (90%) + 1000°C/1h	FCC+B2		8.3×10^{-4}	844	1175	22.0	23.0	10
$AlCoCrFeNi_{2.2}$	AC	FCC+B2		1×10^{-3}	545	1120	20.5	20.5	11

$Al_{0.7}CoCrFe_2Ni$	AC	FCC+BCC+B2		2×10^{-4}	866	1223	7.9	7.9	12
$Al_{0.17}CoCrFeNiTi_{0.09}$	AC + 1200°C/4h	FCC		1×10^{-3}	185	503	58.0	67.0	13
	AC + 1200°C/4h + CR (70%) + 650°C/4h/WQ	FCC+$L1_2$+Ni_2AlTi		1×10^{-3}	1005	1273	15.8	17.0	13
AlCoCrCuFeNi	AC	BCC+2FCC		1×10^{-3}	790	790	0.2	0.2	14
	AC + 960°C/50h + multi-step forged at 950°C (1000%)	BCC+2FCC+σ		1×10^{-3}	1040	1170	1.0	1.0	14
$Al_{0.5}CoCrCuFeNi$	AC + 1000°C/6h/WQ + CR (80%) + 900°C/10min	2FCC		1×10^{-3}	1021	1030	12.0	15.3	15
	AC + 1000°C/6h/WQ + CR (80%) + 900°C/300min	2FCC		1×10^{-3}	610	780	25.6	28.0	15
$Al_{0.2}CoCrCu_{0.2}FeNi_2$	AC + 1200°C/24h + CR (93%) + 700°C/20h/WQ	FCC+$L1_2$	4.49	1.7×10^{-3}	719	1048	26.3	30.4	16
	AC + 1200°C/24h + CR (93%) + 800°C/1h/WQ	FCC	4.51	1.7×10^{-3}	460	732	29.4	31.7	16
$Al_{0.4}CoCrFeMnNi$	AC	FCC		1×10^{-3}	242	529	42.0	47.2	17
$Al_{0.6}CoCrFeMnNi$	AC	FCC+BCC+B2		1×10^{-3}	832	1174	7.7	7.7	17
$Al_{0.5}CoCrFeMnNi$	AC + 1200°C/6h/WQ + CR (78.6%) + 1200°C/5min/WQ	FCC	100	1×10^{-3}	278	619	49.6	60.4	18
	AC + 1200°C/6h/WQ + CR (78.6%) + 1100°C/10min/WQ	FCC+B2		1×10^{-3}	409	755	34.8	43.9	18
	AC + 1200°C/6h/WQ + CR (78.6%) + 1000°C/15min/WQ	FCC+B2		1×10^{-3}	730	968	25.1	29.1	18
$Al_{0.07}Co_{0.29}Fe_{0.29}Ni_{0.29}Ti_{0.07}$	AC + 1150°C/2h/WQ + CR (65%) + 1150°C/1.5m/WQ + 780°C/4h/WQ	FCC+$L1_2$	40-50	1×10^{-3}	1000	1500	50.0	45.0	19
$Al_{0.11}Co_{0.22}Fe_{0.09}$ $Ni_{0.44}Ti_{0.12}$ $B_{0.02}$	AC + 1050°C/12h/WQ + CR (72%) / IA (1050°C/30m/AC) per 10% reduction	FCC+$L1_2$	11	1×10^{-3}	1040	1611	25.0	25.0	20
$Al_{0.5}CrCuFeNi_2$	AC + CR (43%)	2FCC		1×10^{-3}	363	500	16.0	16.0	21

	AC + CR (43%) + 900°C/24 h	BCC+FCC+ $L1_2$		1×10^{-3}	704	1088	5.6	5.6	21
$AlCrFe_2Ni_2$	AC	FCC+BCC+B2		1×10^{-3}	796	1437	15.7	15.7	22
$Al_{0.6}CrFe_2Mn_{1.2}Ni_{0.8}$	AC	BCC+B2		1×10^{-3}	750	880	2.5	2.5	23
$Al_{0.7}Cr_{0.5}Fe_{3.6}Mn_{3.1}Ni$	AC	FCC	123	5×10^{-4}	170	375	40.0	40.0	24
	AC + CR (70%) + 800°C/8h	FCC+B2	5	5×10^{-4}	416	530	25.0	30.0	24
$Al_{0.7}Cr_{0.5}Fe_{3.6}Mn_{3.1}NiC_{0.09}$	AC	FCC	118	5×10^{-4}	380	870	48.0	48.0	24
	AC + CR (70%) + 1000°C/1h	FCC+B2+$M_{23}C_6$+M_7C_3	5	5×10^{-4}	557	1050	25.0	26.0	24
$Al_{0.7}Fe_2Mn_{1.8}Ni$	AC	FCC+B2		5×10^{-4}	270	580	23.0	23.0	25
	AC + 727°C/1h	FCC+B2		5×10^{-4}	420	780	22.0	26.0	25
$Al_{0.7}Fe_2Mn_{1.8}NiC_{0.07}$	AC	FCC+MS		5×10^{-4}	260	680	39.3	39.3	25
	AC + 727°C/1h	FCC+MS+B2		5×10^{-4}	540	875	15.0	18.0	25
	AC + CR (70%) + 1100°C/4h + CR (50%) + 1000°C/4h	FCC+MS+B2	4.5	5×10^{-4}	426	945	32.8	36.0	25
CoCrFeNi	AC	FCC		1×10^{-3}	140	488	76.0	83.0	26
	AC + 1000°C/24h	FCC		1×10^{-3}	130	458	78.0	87.0	26
	AC + 1000°C/24h/SC + CR (80%) + 625°C/1h/SC	FCC		7.3×10^{-4}	540	786	38.0	49.3	27
$CoCrFeNiTi_{0.2}$	AC + 1100°C/5h + CR(80%) + 1100°C/1h + 800°C/1h	FCC+$L1_2$		1×10^{-3}	700	1200	32.0	37.0	28
$(CoCrFeNi)_{94}Ti_2Al_4$	AC + 1200°C/4h + CR(30%) + 1000°C/2h + 800°C/1h	FCC+$L1_2$		1×10^{-3}	625	1080	35.0	38.0	29

CoCrFeNiMn	AC	FCC		1×10^{-3}	215	491	57.0	71.0	26
	AC + 1000°C/24h	FCC		1×10^{-3}	162	443	56.0	68.0	26
	AC + 1100°C/2h + 1000°C/HF +CR + 650°C/30min/WQ	FCC	0.503	1×10^{-3}	888	984	21.0	34.0	30
	AC + 1100°C/2h + 1000°C/HF +CR + 1100°C/30min/WQ	FCC	35.1	1×10^{-3}	300	679	45.3	60.3	30
$Co_{1.4}CrFeMnNi$	AC + 1000°C/24h/SC + CR (80%) + 1100°C/1h	FCC	60-80	7.3×10^{-4}	134	414	66.5	73.5	27
	AC + 1000°C/24h/SC + CR (80%) + 625°C/1h	FCC	1-2	7.3×10^{-4}	586	715	24.3	32.8	27
$CoCr_{1.3}FeMnNi_{0.7}$	AC + 1000°C/24h/SC + CR (80%) + 1100°C/1h	FCC	60-80	7.3×10^{-4}	162	462	50.1	51.6	27
	AC + 1000°C/24h/SC + CR (80%) + 675°C/1h	FCC+σ	1-2	7.3×10^{-4}	1153	1187	1.7	1.8	27
$Co_{0.19}Cr_{0.08}Fe_{1.58}Mn_{1.08}Ni$	AC + 900°C HR (50%) + 1200°C/2h/WQ	FCC	24	2.5×10^{-3}	95	375	45.0	58.0	31
	AC + 900°C HR (50%) + 1200°C/2h/WQ + CR (64%) + 900°C/10min	FCC	12	2.5×10^{-3}	240	645	45.0	59.0	31
$CoCrFeMnNiC_{0.05}$	SLM/scan speed 200 mm/s	$FCC+M_{23}C_6$		1×10^{-3}	829	989	15.2	24.3	32
	SLM/scan speed 600 mm/s	$FCC+M_{23}C_6$		1×10^{-3}	741	874	18.6	39.7	32
$Co_{0.1}Cr_{0.1}Fe_{0.4}Mn_{0.4}$	AC	FCC		1×10^{-3}	213	471	49.0	58.0	33
$Co_{0.1}Cr_{0.1}Fe_{0.4}Mn_{0.4} + C_{2.2}$	AC	FCC		1×10^{-3}	310	650	61.0	63.0	33
$Co_{0.1}Cr_{0.1}Fe_{0.4}Mn_{0.4} + C_{3.3}$	AC	FCC		1×10^{-3}	422	787	76.0	78.0	33
$Co_{0.1}Cr_{0.1}Fe_{0.4}Mn_{0.4} + C_{4.4}$	AC	FCC		1×10^{-3}	467	836	53.0	55.0	33
$CoCrFeMo_{0.1}Ni$	AC + 500°C/4h	FCC		1×10^{-3}	199	479	46.9	51.1	34
$CoCrFeMo_{0.2}Ni$	AC + 500°C/4h	FCC		1×10^{-3}	255	590	52.0	55.1	34
$CoCrFeMo_{0.3}Ni$	AC + 500°C/4h	FCC+σ		1×10^{-3}	305	710	42.5	49.3	34

	AC + CR (60%) + 850°C/1h	FCC+σ+μ		1×10^{-3}	816	1187	18.9	18.9	34
	AC + CR (60%) + 950°C/5 h	FCC+σ+μ		1×10^{-3}	647	1042	30.0	32.5	34
$Co_{1.75}Cr_{0.75}FeMo_{0.5}Ni$	AC + 1200°C/48h/SC + 1100°C HR & CR (70%)	FCC		1×10^{-3}	350	720	21.9	21.9	35
$Co_{2.125}Cr_{0.625}FeMo_{0.25}Ni$	AC + 1200°C/48h/SC + 1100°C HR & CR (70%)	FCC		1×10^{-3}	220	540	57.5	60.0	35
	AC + 1200°C/48h/SC + 1100°C HR & CR (70%) + 800°C/1h/AC	FCC+μ		1×10^{-3}	1311	1410	9.5	12.1	35
	AC + 1200°C/48h/SC + 1100°C HR & CR (70%) + 1000°C/1h/AC	FCC+μ		1×10^{-3}	799	1127	26.0	28.2	35
	AC + 1200°C/48h/SC + 1100°C HR & CR (70%) + 1150°C/1h/AC	FCC+μ		1×10^{-3}	350	918	60.0	62.4	35
$Co_{0.32}Cr_{0.23}FeNi_{0.13}Mo_{0.14}$	AC + 1250°C/6h/WQ + CR (79%) + 1200°C/60min/WQ	FCC	120.6	1×10^{-3}	296	693	57.0	63.2	36
	AC + 1250°C/6h/WQ + CR (79%) + 900°C/60min/WQ	FCC+μ	3.2	1×10^{-3}	711	1096	22.0	38.3	36
$Co_{0.32}Cr_{0.23}FeNi_{0.18}Mo_{0.09}$	AC + 1250°C/6h/WQ + CR (79%) + 1200°C/60min/WQ	FCC	136.5	1×10^{-3}	231	638	75.0	87.2	36
	AC + 1250°C/6h/WQ + CR (79%) + 1000°C/60min/WQ	FCC	36.3	1×10^{-3}	301	718	63.0	77.5	36
	AC + 1250°C/6h/WQ + CR (79%) + 900°C/60min/WQ	FCC+μ	4.4	1×10^{-3}	443	840	42.0	61.6	36
CoNiV	AC + 1200°C/24h/WQ + CR (75%) + 1000°C/60min/WQ	FCC	2.0	1×10^{-3}	517	1049	50.0	55.0	37
	AC + 1200°C/24h/WQ + CR (75%) + 900°C/60min/WQ	FCC	5.6	1×10^{-3}	767	1221	41.0	46.0	37
	AC + 1200°C/24h/WQ + CR (75%) + 900°C/1min/WQ	FCC	27.8	1×10^{-3}	991	1359	31.0	38.0	37
HfNbTiZr	AC + 1300°C/6h/SC	BCC		1×10^{-3}	879	969	14.0	14.9	38
HfTaTiZr	AC	BCC		1×10^{-3}	1356	1452	2.5	4.0	39

$HfTa_{0.6}TiZr$	AC	BCC		1×10^{-3}	750	1110	20.0	22.1	39
HfNbTaTiZr	AC + HIP@1200°C/207MPa/2h + 1200°C/24h + CR (86.4%)	BCC		1×10^{-3}	1202	1295	2.5	4.7	40
	AC + HIP@1200°C/207MPa/2h + 1200°C/24h + CR (86.4%) + 1000°C/2h/SC	BCC		1×10^{-3}	1145	1262	9.7	9.7	40
$Hf_{0.5}Nb_{0.5}Ta_{0.5}Ti_{1.5}Zr$	AC	BCC		1×10^{-3}	903	990	17.0	18.8	41

*Note that the following abbreviations are used in Table S1: AC (as-cast), CR (cold-rolled), HR (hot-rolled), HF (hot forged), UF (upset forged), SC (slow cooled), WQ (water quench), AC (air cooling), IA (intermediate annealing), HIP (hot isostatic pressing), SLM (selective laser melting), FSP (friction stir processing), and GS (grain size).

Supplementary References

1. Komarasamy, M., Kumar, N., Tang, Z., Mishra, R. & Liaw, P. Effect of microstructure on the deformation mechanism of friction stir-processed $Al_{0.1}$CoCrFeNi high entropy alloy. *Mater. Res. Lett.* **3**, 30-34 (2015).

2. Hou, J., Zhang, M., Yang, H. & Qiao, J. Deformation behavior of $Al_{0.25}$CoCrFeNi high-entropy alloy after recrystallization. *Metals* **7**, 111 (2017).

3. Shun, T.-T. & Du, Y.-C. Microstructure and tensile behaviors of FCC $Al_{0.3}$CoCrFeNi high entropy alloy. *J. Alloys Compd.* **479**, 157-160 (2009).

4. Ma, S. *et al.* Superior high tensile elongation of a single-crystal $CoCrFeNiAl_{0.3}$ high-entropy alloy by Bridgman solidification. *Intermetallics* **54**, 104-109 (2014).

5. Rao, J. C. *et al.* Secondary phases in AlxCoCrFeNi high-entropy alloys: An in-situ TEM heating study and thermodynamic appraisal. *Acta Mater.* **131**, 206-220 (2017).

6. Shukla, S. *et al.* Hierarchical features infused heterogeneous grain structure for extraordinary strength-ductility synergy. *Mater. Res. Lett.* **6**, 676-682 (2018).

7. Li, D. *et al.* High-entropy $Al_{0.3}$CoCrFeNi alloy fibers with high tensile strength and ductility at ambient and cryogenic temperatures. *Acta Mater.* **123**, 285-294 (2017).

8. Niu, S. *et al.* Strengthening of nanoprecipitations in an annealed $Al_{0.5}$CoCrFeNi high entropy alloy. *Mater. Sci. Eng. A* **671**, 82-86 (2016).

9. Wani, I. S. *et al.* Ultrafine-Grained $AlCoCrFeNi_{2.1}$ Eutectic High-Entropy Alloy. *Mater. Res. Lett.* **4**, 174-179 (2016).

10. Wani, I. S. *et al.* Tailoring nanostructures and mechanical properties of $AlCoCrFeNi_{2.1}$ eutectic high entropy alloy using thermo-mechanical processing. *Mater. Sci. Eng. A* **675**, 99-109 (2016).

11. Lu, Y. *et al.* Directly cast bulk eutectic and near-eutectic high entropy alloys with balanced strength and ductility in a wide temperature range. *Acta Mater.* **124**, 143-150 (2017).

12. Wang, Q. *et al.* A cuboidal B2 nanoprecipitation-enhanced body-centered-cubic alloy $Al_{0.7}CoCrFe_2Ni$ with prominent tensile properties. *Scr. Mater.* **120**, 85-89 (2016).

13. He, J. *et al.* A precipitation-hardened high-entropy alloy with outstanding tensile properties. *Acta Mater.* **102**, 187-196 (2016).

14. Kuznetsov, A. V., Shaysultanov, D. G., Stepanov, N. D., Salishchev, G. A. & Senkov, O. N. Tensile properties of an AlCrCuNiFeCo high-entropy alloy in as-cast and wrought conditions. *Mater. Sci. Eng. A* **533**, 107-118 (2012).

15. Tsai, C. W. *et al.* Microstructure and tensile properties of $Al_{0.5}CoCrCuFeNi$ alloys produced by simple rolling and annealing. *Mater. Sci. Technol.* **31**, 1178-1183 (2015).

16. Wang, Z. G. *et al.* Effect of coherent $L1_2$ nanoprecipitates on the tensile behavior of a FCC-based high-entropy alloy. *Mater. Sci. Eng. A* **696**, 503-510 (2017).

17. He, J. *et al.* Effects of Al addition on structural evolution and tensile properties of the FeCoNiCrMn high-entropy alloy system. *Acta Mater.* **62**, 105-113 (2014).

18. Park, J. M. *et al.* Effect of annealing heat treatment on microstructural evolution and tensile behavior of $Al_{0.5}CoCrFeMnNi$ high-entropy alloy. *Mater. Sci. Eng. A* **728**, 251-258 (2018).

19. Yang, T. *et al.* Multicomponent intermetallic nanoparticles and superb mechanical behaviors of complex alloys. *Science* **362**, 933 (2018).

20. Yang, T. *et al.* Ultrahigh-strength and ductile superlattice alloys with nanoscale disordered interfaces. *Science* **369**, 427 (2020).

21. Ng, C. *et al.* Phase stability and tensile properties of Co-free $Al_{0.5}CrCuFeNi_2$ high-entropy alloys. *J. Alloys Compd.* **584**, 530-537 (2014).

22. Dong, Y., Gao, X., Lu, Y., Wang, T. & Li, T. A multi-component $AlCrFe_2Ni_2$ alloy with excellent mechanical properties. *Mater. Lett.* **169**, 62-64 (2016).

23. Shaysultanov, D. G. *et al.* Novel $Fe_{36}Mn_{21}Cr_{18}Ni_{15}Al_{10}$ high entropy alloy with bcc/B2 dual-phase structure. *J. Alloys Compd.* **705**, 756-763 (2017).

24. Wang, Z., Baker, I., Guo, W. & Poplawsky, J. D. The effect of carbon on the microstructures, mechanical properties, and deformation mechanisms of thermo-mechanically treated $Fe_{40.4}Ni_{11.3}Mn_{34.8}Al_{7.5}Cr_6$ high entropy alloys. *Acta Mater.* **126**, 346-360 (2017).

25. Wang, Z. & Baker, I. Effects of annealing and thermo-mechanical treatment on the microstructures and mechanical properties of a carbon-doped FeNiMnAl multi-component alloy. *Mater. Sci. Eng. A* **693**, 101-110 (2017).

26. Salishchev, G. *et al.* Effect of Mn and V on structure and mechanical properties of high-entropy alloys based on CoCrFeNi system. *J. Alloys Compd.* **591**, 11-21 (2014).

27. Zaddach, A. J., Scattergood, R. O. & Koch, C. C. Tensile properties of low-stacking fault energy high-entropy alloys. *Mater. Sci. Eng. A* **636**, 373-378 (2015).

28. Tong, Y. *et al.* Outstanding tensile properties of a precipitation-strengthened $FeCoNiCrTi_{0.2}$ high-entropy alloy at room and cryogenic temperatures. *Acta Mater.* **165**, 228-240 (2019).

29. He, J. Y. *et al.* Precipitation behavior and its effects on tensile properties of FeCoNiCr high-entropy alloys. *Intermetallics* **79**, 41-52 (2016).

30. Sun, S. *et al.* Enhanced strength and ductility of bulk CoCrFeMnNi high entropy alloy having fully recrystallized ultrafine-grained structure. *Mater. Des.* **133**, 122-127 (2017).

31. Yao, M., Pradeep, K. G., Tasan, C. C. & Raabe, D. A novel, single phase, non-equiatomic FeMnNiCoCr high-entropy alloy with exceptional phase stability and tensile ductility. *Scr. Mater.* **72**, 5-8 (2014).

32. Park, J. M. *et al.* Superior tensile properties of 1%C-CoCrFeMnNi high-entropy alloy additively manufactured by selective laser melting. *Mater. Res. Lett.* **8**, 1-7 (2020).

33. Chen, L. *et al.* Heavy carbon alloyed FCC-structured high entropy alloy with excellent combination of strength and ductility. *Mater. Sci. Eng. A* **716**, 150-156 (2018).

34. Liu, W. H. *et al.* Ductile $CoCrFeNiMo_x$ high entropy alloys strengthened by hard intermetallic phases. *Acta Mater.* **116**, 332-342 (2016).

35. Ming, K., Bi, X. & Wang, J. Precipitation strengthening of ductile $Cr_{15}Fe_{20}Co_{35}Ni_{20}Mo_{10}$ alloys. *Scr. Mater.* **137**, 88-93 (2017).

36. Bae, J. W. *et al.* Effect of μ-precipitates on the microstructure and mechanical properties of non-equiatomic CoCrFeNiMo medium-entropy alloys. *J. Alloys Compd.* **781**, 75-83 (2019).

37. Sohn, S. S. *et al.* Ultrastrong Medium-Entropy Single-Phase Alloys Designed via Severe Lattice Distortion. *Adv. Mater.* **31**, e1807142 (2019).

38. Wu, Y. *et al.* A refractory $Hf_{25}Nb_{25}Ti_{25}Zr_{25}$ high-entropy alloy with excellent structural stability and tensile properties. *Mater. Lett.* **130**, 277-280 (2014).

39. Huang, H. *et al.* Phase-transformation ductilization of brittle high-entropy alloys via metastability engineering. *Adv. Mater.* **29**, 1701678 (2017).

40. Senkov, O. & Semiatin, S. Microstructure and properties of a refractory high-entropy alloy after cold working. *J. Alloys Compd.* **649**, 1110-1123 (2015).

41. Sheikh, S. *et al.* Alloy design for intrinsically ductile refractory high-entropy alloys. *J. Appl. Phys.* **120**, 164902 (2016).
